# Dino’s Story: The Challenges of Co-writing the Life History of a Brazilian Gangster

**DOI:** 10.1007/s10612-025-09852-1

**Published:** 2025-12-09

**Authors:** Corentin Cohen

**Affiliations:** Department of Politics and International Relations, Manor Road, Oxford, OX1 3UQ UK

## Abstract

This article reflects on my attempt to co-write the life history of Dino, a former member of two gangs in São Paulo, Brazil. While introducing constraints, co-writing prolonged my ethnographic fieldwork, and enhanced reflexivity by allowing access to the rewritings and translations that Dino elaborated, which led to a better understanding of what telling his story meant to him and what he thought was important about it, thereby revealing important elements of his gangster identity, but also of racial dynamics of gangs. These led to our fictionalizing his story, which—in contrast to much research on gangs in Latin America that is often characterized by a fundamental inequality between researchers and their interlocutors—allowed for a fairer and more transparent ethnographic “pact” between Dino and myself. This allowed us to both preserve our ethical senses of self and to maintain our relationship, and improved the life history that we crafted together.

## Introduction

This article is a reflection on my attempt to co-write a fictionalized version of the life history of Dino,^1^ a former member of two different gangs in São Paulo, Brazil. Although fictionalization and co-writing are not uncommon in the social sciences, my initial endeavors immediately raised a number of questions. “When is [something] fictionalized?”, asked one of the reviewers of the first version of this article, “what does [fictionalization] even mean? Don’t we all do this all the time? I have been splitting people to create unidentifiable actors—e.g. (name) did this, (name) did that—but they are actually the same person. So, I am wondering how different this is from what we normally do”. Similarly, a participant at the ERC GANGS project workshop on “Gang Lives”, where I first introduced the life history, commented “if this is co-written, what is co-invented and what is not, and whose voice are we hearing?”.

Such questions can be read as raising issues linked to the “veracity” of the research process, something that has long been a matter of debate in the social sciences (see Blondet and Lantin Mallet 2017). What I want to consider instead in this article is how co-writing Dino’s life history as a fiction led me to question the whole process of writing about gangs and gangsters, and to confront certain critical epistemological blind spots. Recent work has highlighted how much social science knowledge and theory owes to research interlocutors whose role is often invisibilized (Rappaport 2008). This is particularly true with regard to authorship and the question of who and what is an author, a discussion that has permeated journals and institutions that have tried to define what constitutes contribution to research and how to recognize this fairly (for a literature review, see Blissett 2024). Gay y Blasco and Hernandez (2020: 167) note in this regard that “writing ethnography most often involves a transformation of the role of informants from active co-creators of anthropological knowledge in the field to ethnographic data to be deployed by the anthropologist as academic authority”. This position seems inherited from the colonial anthropologist credited with writing theory and producing knowledge about others but erasing their contributions and theorizations (Strang 2006).

Drawing on my experience of collaborating with Dino, I will show how certain constraints linked to his particular moral codes and ethics, as well as his strategies for presenting himself, forced me to reflect on my own biases and led to a negotiation, the outcome of which was our co-writing a fictionalized version of his life history. This offered two major advantages. First, both co-writing and thinking about the fictionalization of Dino’s life history prolonged the ethnographic fieldwork experience. It was particularly revealing to observe and be able to consciously ponder upon the different rewritings of Dino’s story, the choices, erasures, translations, and different narratives, especially in relation to traumatic, violent, or illegal issues. Co-writing a fictional account thus allowed us to craft together a mutually reflexive account of Dino’s trajectory.

Second, co-writing Dino’s life history as a fiction allowed us to develop a new ethical approach and constitute a fairer relationship between us as researcher and research interlocutor. It is now well established that social science research is often characterized by a fundamental inequality between researchers and their interlocutors. This is often especially true of gang research, where interlocutors frequently have less chances and ability to express their visions of the world in public outlets as a result of the underworld nature of their lives and activities, as well as broader social inequalities. In the case presented in this article, the co-writing of a fictionalized account of Dino’s life was a step towards greater transparency and accountability between him and I. It established a new ethnographic “pact” that allowed us to maintain a relationship with divergent senses of ethics, while at the same time producing something that we believe substantively advances our understanding of São Paulo’s gangs, including in particular the history of their racial dynamics.

This article is single authored. It constitutes my own reflections on our process of co-writing. The fictional life history that Dino and I crafted together will be coauthored and published elsewhere. In what follows, I begin by discussing what we understand as social scientific writing—how we do it, what is reality and what is fiction, and for which audience we write—before then explaining how the ethnographic pact that Dino and I came up with influenced the representation of his life history we crafted together. This pact allowed me to observe how Dino wanted his story to be recorded and remembered, and what he agreed to put on paper. This offered space for a Goffmanian analysis of his self-presentation and performance in relation to an imaginary readership. I also got access to his interpretations and beliefs at the time certain events occurred, and what he thought about them now. In between these gaps, lay opportunities to interpret and revise my observations, not just about Dino, but about gangs.

The first section below introduces my motivations for researching Dino’s life, and particularly my interest in focusing on his social mobility, and how this would have led me to craft biased representations of his trajectory had I not shown him my first drafts. In the second section I analyze how Dino’s concern with his street status and his moral views constrained the writing, but also limited his ability to tell his own story. I then highlight and explore the fundamentally different perspectives on what writing means for a gangster and for an academic researcher, and discuss how we engaged in co-writing practically, the approach we choose in relation to thinking about imaginary readerships, what we expected to get out of our co-writing, and how fictionalization emerged as the key to crafting his life history. In the final section, I present the new ethnographic pact that Dino and I crafted together in order to write a fictionalized narrative about an imaginary alter ego. This improved both my understandings of Dino and gangs, as well as, more broadly, the kind of story we told.

This was very much an experimental and highly ad hoc process during which our discussions regularly led us to redefine and adjust our ambitions and methods to write Dino’s life story. My hope is that by being explicit about the underpinnings of this experiment, it will inspire further discussion and research. In many ways, however, this article raises more questions than it answers. The conclusion tries to pull together some of these, in particular with regard to how we can co-write in an ethical way, fictionalization, and why co-writing and fictionalization need to be part of a longer-term, longitudinal ethnographic fieldwork.

## My Expectations when Narrating Dino’s Life History

In this first section, I reflect on the motivations that lead ethnographers to write about the lives of gangsters. While I initially thought I was just interested in understanding gangs and what I considered to be an important and under researched part of the history of São Paulo’s criminal world, I was equally influenced by a certain number of biases that became explicit in the process of co-writing. These made me adopt a certain position and influenced how I wanted to tell Dino’s story, as I will now detail.

I met Dino in 2014 through some of my research interlocutors, and he became one of the brokers for my research. Dino helps youths “at risk” of becoming involved with drug trafficking and tries to keep them busy with cultural and artistic activities. After spending time discussing the issue of crime and its history in São Paulo, he put me in touch with a number of delinquent youths and former petty criminals, and even gave me tips and advice as to how to interview them. In the year that followed, we shared ideas about what was going on in the city, and on some of the topics I researched: the narratives and representations of violence in the media and popular culture, criminal governance, and the influence of the *Primeiro Commando da Capital* (hereafter PCC) organized crime group in the city. I observed him at work, attended meetings and informal encounters with offenders involved or at risk of getting involved in drug trafficking. Dino was always generous and supportive. I could see he had a certain pride in enabling my research.

Up until 2018, I spent time with him, I got to know his friends, and his family, but he was always discreet about himself. I only really knew the bare bones of his life trajectory, which began when Dino was seven and lost his mother, who was “black”—Dino emphasized—and “had loved him very much”. He was then abandoned by his father and adopted by another family, whom he said was “white and very Catholic”. Dino ran away from this adoptive family and left school to live on the street. This is how he became involved in a gang that operated in the area, called the *Biscoitos* (Biscuits). Dino learned to steal, fight, and make it in the street with the Biscoitos. Dino’s life took a new direction when he fell in love with a woman who was involved in theatre. Although he remained in the gang, as part of his attempt to seduce her he tried to develop an artistic career as an actor and playwright. This quest for a new life led him to join black social movements and awareness programs. Later, however, Dino joined a second gang that would legally be defined in Brazil as a *quadrilha*, a criminal association. It was then that Dino got involved in armed robbery. He ended up being caught and spending some time in prison, before further developing his career as an actor, playwright, and social activist.

Although I had been aware of Dino’s circulation between these very different worlds, I had not been too sure how it had happened and what this meant for him. This triggered my initial interest in developing a better understanding of his life history. Dino’s social mobility made him stand out in relation to most of my other research interlocutors who had less extensive networks and access to other social and cultural worlds. His mobility seemed to reflected an easy understanding of the multiple worlds he had crossed; in other words, Dino came across as something of a social chameleon. I had seen him engaging with people from different milieus and communities, changing tone, cultural references and styles depending on who he was speaking with. My hope was to discover more about these relationships that I thought were more interesting than any other aspect of his life. As I would understand later, this was very much a projection of my personal research interests. In actual fact, Dino’s mobility was more contentious, less fluid, and tenser than I had understood it—and that he projected.

When I started to think about writing a gang member’s life story for the ERC GANGS project, I looked for someone I had previous experience of working with, whom I had observed, and who I was close enough with to present a draft of the life history in order to discuss it. In this regard, Dino already wrote plays and had a career as an actor. I believed this would facilitate the process and render it more interesting. My intuition about the potential for collaborating seemed to be confirmed by Dino’s positive reaction when I introduced him to the whole idea of writing gangsters’ life stories to him—he said it would be amazing to write several! I gave him a translated version of the ERC GANGS project proposal, and we talked about other famous life histories, including Maria Carolina de Jesus’ (2020) famous *Quarto de Despejo*, and I translated excerpts of Robert Gay’s (2015) landmark *Bruno: Conversations with a Brazilian Drug Dealer* for him. Inspired by this latter book, I prepared questions about his trajectory in relation to different micro-, meso-, and macro-level social dynamics with the intention of discussing how they had affected Dino’s life, in order to gather as much information as possible and fill the gaps in my knowledge about his trajectory.

While reading about Bruno, however, Dino focused mainly on trying to guess who he really was, which triggered a concern about the limits of anonymization and of how much he wanted to tell. This also prompted a discussion on the role of families, and the connections between police and gangs. This initially seemed promising. These relationships are well known in Rio de Janeiro, going back into the past through to the present, but less so in São Paulo, when the role played in urban developments by gangs is often minimized or ignored, at least historically. There are accounts of lynchings, vigilantes, and other forms of urban violence (Bicudo 1988; Sinhoretto 2009), as well as social movements including youth movements, but there are no accounts of the role that gangs and groups of street children played in urban dynamics in the 1980s and 1990s. Dino and I decided to therefore carry out a series of interviews about his life during this period that I would later draw on in order to write something that was still undefined but that he would then comment on. In this regard, I have to admit that I still thought about carrying out the interview—the data collection—and the writing as two disconnected processes. My engagement with Dino very quickly revealed this to be a fallacy and it is what led to us engaging in a much more extensive process of co-writing, as the next section highlights.

## How My Interlocutor’s Ethos, Moral and Ethics Influenced Our Co-writing

At the heart of the matter were Dino’s morals and sense of ethics, which very much constrained his self-presentation. In particular, his active embodiment of what could be called “the code of the street” (Anderson 1999) and his concern with maintaining “*postura*”—literally, “posture”, but closer to meaning “presentation”—meant that things had to be said in a particular way, as well as certain events occulted or only discussed in a specific way. I felt, however, that my role was to point out gaps and inconsistencies in his narrative, and bring them to light. This seemed important to me, as I was coming to understand that Dino was perhaps not as successful in circulating different social worlds as I had initially imagined. He presented his venturing into the world of theatre and arts as an attempt to leave the gang and escape from a life of crime. Yet, one of the things he mentioned in passing was the fact that he had partly funded his first play with proceedings from his illegal activities. In other words, the two spheres were much more intertwined than he cared to admit.

Even if when we had open and comprehensive discussion about such inconsistencies, Dino would consistently provide less information when my recorder was on. He would complement his explanations or even sometimes contradict what he had said after recording sessions, thereby revealing a gap between what he would share with me and what he would accept to have written up. I initially believed that his hesitancy and contradictory narratives were due to the potential impact he thought the story might have vis-à-vis his family, or on the young people he worked with as a social activist. I thought that by pseudonymizing individuals and changing certain details about events things could be kept anonymous, but Dino was not more forthcoming when I talked about these with him. It took me a while to understand that this was not about trust, or at least not about not trusting me. Rather, Dino distinguished between what he told me and what he felt could be put in the public domain without betraying his perceived moral obligations vis-à-vis his former gangster peers. The latter was simply not acceptable for him, and this was what made my control over the narrative problematic. Dino wanted to be in control. This was how he had survived the streets, the gangs, prison, and continued to help the youths who trusted him.

Part of Dino’s concern stemmed from the idea that secrets were a form of protection, and that they should never be betrayed. During my own fieldwork in the streets of Sao Paulo, I had often heard that “secrets save lives”, a saying that came in the form of a warning, but also as a motto, that the group shared and that tied them together. I had found it natural when Dino asked me to turn off the recorder to tell me his stories. This was because it was his voice, his responsibility. I also understood that he avoided talking about certain topics when the recorder was on (e.g. names of people, but also about weapons, money, or specific *quadrilha* actions). Indeed, he articulated that he was providing me with both “frontstage” and “backstage” conversations, with the former only being “por ingleses ver” (for English-speakers). The problem was that I felt that if I wanted to show the unacknowledged relevance of São Paulo’s gangs for the city’s dynamics in the 1980s and 1990s, too much was having to be left out. Even worse, in a discussion that occurred in the middle of the interview process, Dino told me that giving the reader too many details about the practices of the gangs could damage his and our credibility, and that we needed to reduce the level of empirical detail. This was the opposite of what I imagined was needed to compose a good gangster life history narrative, at least for an academic readership.

At the same time, however, Dino’s behavior reflected how Sao Paulo’s underworld has been heavily influenced by the PCC’s particular ethical code. This is very much relational and ideal. It emphasizes the positioning and attitudes of individuals much more than any positively defined body of laws or rules (Biondi 2014) that constitute a normative and moral order (Feltran 2020). As such, it constrains how anything can be said and what can be done. Dino later explained that saying too much would cast doubt on “the honor and reputation of his character”, even if he remained anonymous. He was scared that informed readers would consider his character as trying to show off and therefore lacking ethics. It felt as if we were being watched by an imaginary and powerful institution, along the lines of Foucault’s (1977) famous analysis of the Panopticon. Not only had Dino internalized the PCC’s norms, but he wanted to use our writing to show he still had street credibility. He referred to the narratives that had been put together by individuals who he regarded as outsiders to the world of crime but who had capitalized on its image in order to advance their careers. He wanted to demonstrate “*postura*” in order to signal his street legitimacy, as individuals who follow “*proceder*” (ethics) are supposed to take care of the words they use, be thoughtful, and must, in all situations, discuss and try to identify what is right and fair.

Partly as a result of this, Dino insisted that his experience with gangs was specific to him. He was always reluctant to generalize or extrapolate from his trajectory. He was not judgmental or dogmatic, but I often had the impression that he wanted to show his experience and seniority in the world of the street. He carefully pictured himself as someone moral, reflective and wise, always well balanced. Indeed, the reason he was such an effective research broker was precisely because of his moral commitment to the code of the street, which meant that he could navigate it and advise me well. But it also meant that he was not completely able to adapt to the ethics of other milieux, and I am not certain that he was able to distance from his street subjectivity despite the years that had passed. This fundamentally constrained our writing and influenced the dynamic of our collaboration. But it also highlighted the existence of critical differences in how and why a former gangster and a researcher write (which might seem obvious, but that researchers often forget).

## What Writing Means: The Problem of Authorship and the Boundaries between Fiction and Reality

When he was telling me about his life during our interviews, Dino often offered vivid details and rich descriptions that I felt really facilitated my work. He would generally answer my questions with long, meandering stories, entertaining vignettes, and would also repeat dialogues in a lively and physical manner that made me feel as if I was directly witnessing scenes that had occurred decades ago. At first, I was delighted to have these as I felt they conveyed a feeling of authenticity that would be hugely important for writing Dino’s life story. At the same time, it rapidly became clear to me that these stories, vignettes, and dialogues, even if they seemed eminently true or at least possible, did not leave space for alternative interpretations.

Dino would often describe things as a stream of consciousness that mixed up his personal experiences and his world views. Some of his stories and vignettes moreover came as long notes, with little or no punctuation, sent via messaging applications. These were often highly compelling and even persuasive. But more than anything else, these texts that he sent me were very much appropriated by him. Dino clearly knew the kind of representation he wanted us to craft together. At first, he imagined that we would use his name in the story and that he would be Brazil’s representative amongst a global cast of gang members’ stories collected for the ERC GANGS project. At the same time, he also wanted to tell potential readers a story of inequality and segregation, while also highlighting the dangers of the gangster career and of drug dealing. I feared that this would lead to our crafting a narrative that would be too normative, even if I subsequently realized that this story would have beautifully illustrated the importance of living according to particular morals and ethics in his life, and how this had shaped his relationships with different social groups.

This was not the path we went down, however, as I actively tried to push him in a different direction, one that was more reflexive and critical. The gap between our ideas of what constituted a “good” life history became evident after my first attempt at organizing a complete narrative of Dino’s life. I saw this very much as a first draft, full of gaps and inconsistencies, but thought it would be a basis upon which to move from carrying out interviews to my writing things up. When he read it, Dino was irritated with my Portuguese mistakes but he had no comments on the content itself. He added a few details to what he thought was a cool story. He said he enjoyed it. To my surprise, he was fine with what I felt were confusing and contradictory elements. I also had the impression at that point that many of the descriptions of elements of his life that I had collected did not reconcile. For instance, Dino had offered several different narratives of how he had come to be a street child and joined a gang. In some of our interviews he heavily insisted that he had not been a victim, because, he said, he had “chosen”—at the age of seven—to leave his (adoptive) family, and proffered an account whereby the gang had constituted a “new family” for him. But during others, he would emphasize events and episodes that led him to conclude that the gang was a predatory institution which exploited younger members.

I was interested to explore how and why these different interpretations were elaborated during the course of his life trajectory. It is something that longitudinal research is particularly confronted with. Drawing on his ethnographic research in the poor urban neighborhood *barrio* Luis Fanor Hernández, in Managua, Nicaragua, Dennis Rodgers (2021) for example discusses the case of Bismarck, a former gang member turned drug dealer turned businessman, who is one of his key research interlocutors, and whom he interviews regularly, and who at one point decided to erase his drug dealing past from his self-narration, mainly due to the increasing stigmatization of drug dealing over time in the neighborhood. Dino was clearly similarly seeking to downplay or leave out elements of his past gang life that he now felt were immoral or unethical acts.

At the same time, the boundaries between Dino’s lived experiences and his narratives about them were also unclear for another reason. In many ways, Dino very much understood what we would call “ethnographic writing” simply as a way of telling stories, and he saw no reason why he should not do the same with his narrative about his life. He was trying to not just control the narrative but also to actively craft it, in order to put forward a particular image of his character. One time he sent me a play that he had written, which he said was inspired by the life of a fellow gang member, but which included a number of the stories he had told me during our interviews were about himself. Looking back, the situation reminds me of the documentary film *The Act of Killing*,^2^ in which aging gangsters who were members of the government-backed death squads involved in the killing of Communists in 1950s Indonesia were interviewed half a century later by the director, Joshua Oppenheimer. They ended up re-enacting the execution of their victims dressed up as their favorite 1940s and 1950s Hollywood gangsters, creating a film within the documentary. This surreal conflation of fiction and reality created the conditions through which they could present themselves as heroes, against predominant interpretations of these past killings, although in some cases it also caused some of them to undergo moral shocks.

## Co-writing and Fictionalizing as a New Ethnographic Pact

As I wrote up initial drafts of his life history for comment, I increasingly felt that Dino considered my function as simply to collect and organize his many stories, and then write them up in a way that could become a book regarding which he would have the final authority. As a researcher, I was motivated to tell a story that was as close as possible to the “truth”, and that had some sociological import. I thought I was supposed to be the guy asking questions and writing a story, and Dino challenged that. At the same time, I did not want to disrespect Dino, and ethically, it felt wrong to write his life history alone. The solution that we worked out was to assume the ambiguity of Dino’s narrative by calling it a fiction and creating an imaginary alter ego for him. If recomposing and fictionalizing were necessarily elements in gangsters’ lives, then why not embrace this in order to understand them better?

To do so, we followed what Jon Wolseth (2019: 345) identifies as a “logical step” in redefining the relationship between the ethnographer and their research interlocutor(s). This is important, he says, as “we construct our ethnographic texts around the relationships we’ve created as a (subjective) participant in the events upon which data collection is based”. Various ethnographers have experimented with different approaches to establish new working relationships with their research interlocutors. Paloma Gay y Blasco and Liria Hernández (2020), for example, recently published a book offering their different voices and analyses of the same events, including of their interactions over the course of 30 years as researcher and research interlocutor, to highlight the “reciprocal game” that their relationship entailed. In a different vein, Tobias Hecht (2006) came to the conclusion that the narrative that Bruna, a former street child and transvestite prostitute, offered him about their life during the course of their interactions was mostly made up or exaggerated, but decided to take what he calls their “ethnographic fictions” seriously as meaningful reflections of their life experiences.

At the same time, composing fictional characters, situations, or dialogues has always been part and parcel of the work of ethnographic research. As Jones and Rodgers (2019) recall, “ethnographers have adopted the use of testimonio, biography and the writing of *quasi-fiction* [my emphasis] to facilitate their craft”. They refer to Wolseth’s study of street kids in the Dominican Republic, *Life on the Malecon* (2014), in which he makes clear that “to aid the flow of the story… characters are sometimes composites of a number of kids I met, and narrative time has been compressed, but the tangibility and plausibility of street life has remained”. As is likely also the case in relation to many other ethnographies, this led Wolseth to invent “scenarios” that selected or cut elements, but always with an eye to remaining “plausible”, or—as is often put—“representative”, based on what he thought he understood from his long-term interactions with his research interlocutors and the Dominican context.

Yet as other contributions to this special issue highlight, there is always a lot that escapes us, even after years of research into the life of one person or of a community, and moving from seeking to represent Dino’s life history as accurately as possible on the basis of unequal exchanges to co-writing a fictionalized version opened a new conceptual space. How did it work? Instead of working on Dino’s stories and interviews by myself, we used them as a canvas to together create a persona that would be Dino’s fictional alter ego, and negotiated a story line to this fictional alter ego’s life trajectory. This very much made co-writing part of the ethnographic process, as we exchanged on how Dino wanted to tell the story, how he wanted it to be recorded, and what he agreed to put on paper. We also looked for translations, for equivalents, we recomposed characters and stories. This was a job clearly better done together, as I could only know indirectly about events that had occurred 30 years ago. This was especially true in a context where I could find no archives or media traces of Dino’s gangs.

During our earlier interviews, I had become frustrated with Dino’s performance and his constant re-writing of things. But the process made me understand that Dino was not (completely) playing me around. Fiction and playing characters were in many ways a coping mechanism for him. Narrating his life story had a different function than just providing material for academic analysis. Since his childhood, Dino liked to impersonate and imitate his family, neighbors, schoolmates, and teachers. He had liked to imagine that he was a TV star, an artist, and often played out small scenes. This had made him popular at school and is what brought him to the world of theatre. Dino was not just omitting information or events. He was using my “research” to elaborate on his own life, in ways that corresponded to his desires and aspirations. Fictional stories and vignettes were his own way of writing his emotions. This brought a new analytical line in the story.

By getting access to Dino’s doubts, his conflicting views and interpretations about life trajectory, I was able to understand him better, as well as gangs more generally. For instance, one thing that Dino wanted to explain through his life history was how gang members who had a family would often leave their gang faster than those living on the streets. He explained this in terms of having a family increasing the chances of mitigating the experience of poverty by “always putting food on the table”. This very much connected with his idea that gangs constituted substitutes for families, something that was also intertwined with his contemporary political engagement. At the same time, he recognized that this belief did not arise from his own life experience, as even if he resented his adoptive family, whom he said he had “chose” to leave, he had come to realize that they had likely done everything they could to get him out of the gang. We were able to use the imaginary alter ego narrator figure in our co-written fictionalized life history to deal with this apparent contradiction and reconcile Dino’s general beliefs about gangs with his own lived experience and the evolutions of his own interpretations about his life, since we could make them have a supportive family in our fictionalized account.

Our “pact” also had other effects, including in particular allowing for race relations to come to the fore in our telling of the history of gangs in 1980s and 1990s São Paulo. This is something that has been mostly absent from research on crime in São Paulo, but which we were able to put into our co-written fictionalized life history because, firstly, Dino was more open to talk about the issue, and secondly because the distancing effect of writing a fictionalized life history enabled him to talk more about his emotions. This generated new insights into his experience of racial tensions in gangs which we were able to put at the center of the co-written fictionalized life history.

The question of race is at the center of political debates in Brazil, in particular with regard to the issue of police violence. In 2022, 76.5% of the 47,508 victims of homicide in the country were black, as were 83% of the 6,430 people killed by the police (Malcher 2023). But race is often set aside in scholarship about criminality in São Paulo. Although it is frequently regarded as an important contextual variable or structure, including in violence between police and (black) criminals, it is rarely actively taken into consideration as a key process fundamentally structuring the criminal world of the city or the PCC.

In this regard, it was striking that Dino had initially completely invisibilized a key racial dimension of the Biscoitos, his first gang, namely that it was a black gang. Many of his Biscoitos stories revolved around fighting another gang, the Razors, for the control of the neighborhood. He’d often explain how the two gangs had different ambitions. Where the Biscoitos fought for survival, the Razors fought for money. The latter’s ambition was to consume, to have stylish shoes and clothes, Dino explained. It took a while for Dino to mention that the Biscoitos had been a black gang, and the Razors a white gang, and I did not initially understand why he had not mentioned it before, especially as he unambiguously defined himself as black, and had (subsequently) been part of black activist movements. Just as surprising was the fact that after first joining these black activist movements, Dino decided to join a second gang in which he was the only black member.

Dino’s choice to ignore the racial dimension in our first discussions was very much in line with contemporary narratives that in the Brazilian world of prison and crime all individuals are equals. This reflects an idealized vision shaped by the advent of the PCC in the early 1990s whose motto became “*Peace*,* Justice*,* Freedom and Equality*”, and they are credited with being the main force driving the drastic decrease in homicides in Sao Paulo during this period. Most of my research interlocutors shared views that the advent of the PCC represented a transition from a brutal and unstable world to a “civilized” one in which violence, barbarity and inequalities of treatment and status of criminals were abolished (Cohen 2022).

Because we focused on a fictional alter ego, co-writing allowed us to opened up a discussion in which Dino was able to express and criticize the racial dynamics that had structured his life experience, but which he chose not to bring to the fore so as not to seem to be disrespecting the race-blind equality narrative that still dominates today. This additionally opened up new discussions about the challenges that he faced when circulating between the milieus of black activism, theatre, and art more generally. The initial story that I had written about Dino’s life had very much focused on how he had pushed against social boundaries, but as we co-wrote a fictionalized version, it became easier for Dino to discuss what he called the “essence” of the worlds he had circulated in, including in particular having to overcome racial and social boundaries.

It’s possible that this reconstruction of racial dynamics could have occurred through conversation. After all, Dino has clear political views about race and racism. He understands police violence, poverty, and segregation as the legacy of slavery, and as ways to control black and working populations. But Dino was less clear when it came to how and when racism had affected his own life, partly because doing so required an emotional opening and space. Dino often expressed the view that he had been a victim of racial segregation and of racism within his (white) adoptive family, but as we developed a racial angle to our co-written fictionalized life history, he began to say that this was nothing more than an excuse he drew on to justify the choices he had made.

In other words, dealing with the question of race required us to explore Dino’s emotions, which he initially downplayed in his narratives and stories about his life, even if he often told me that as an artist, he was very sensitive. This was less the case when he self-presented as a gangster, however. But when we talked about racism in the world of gangs, this generated significant emotions in Dino, especially when we co-wrote the (true and imagined) stories of each of the Biscoitos gang members. In stark contrast to the trajectories of the white gangsters whom Dino knew, the overwhelming majority of his fellow black gang members were all dead or had ended up in prison, and Dino’s imaginary alter ego whose life story we were co-writing was the last survivor of the Biscoitos. This was something that Dino clearly found upsetting, particularly as the surviving (white) members of the Razors gang had all climbed the criminal ladder, often managing to avoid prison due to family relations with police officers. The fictionalized life history very much puts the importance of such racial dynamics over the long-term at its core. The racial identifications, references, and analysis in the final story will be based on Dino’s. But they were elaborated along my questions about the meaning of identity and race in different contexts, and how he had positioned himself in relation to such processes during his life.

The emotionality that our new ethnographic pact allowed also meant that Dino became more open to talking about the long-term trauma associated with the effects of violence, something that I had found difficult to discuss before. As Johnny Steinberg (2015) writes in his biography of Assad, a Somali civil war refugee trying to make his way and find peace in South Africa, despite developing a real sense of complicity with Assad over many hours of interviews, there were always some questions—about relatives, intimacy, or life changing decisions—that he never felt able to ask, out of respect, as well as some that he regretted having asked, due to the emotions these raised. I sometimes felt in a similar situation during my research with Dino. One time, for example, in response to a question about how his family felt about his having been a gang member, he told me that he had once suffered a major humiliation when his family tried to force him out of the gang. I could see tears well up in his eyes, and I decided not to press him on the issue. But it came up again as we engaged in the process of co-writing, as we discussed what could have happened to his alter ego that would have permanently separated him from his family. He answered sadly that “the mother could call an uncle or a relative, a figure of authority, to be strict and give him a lesson, a physical one”. At this point, though, the sadness I had sensed in his voice gave way to a great deal of anger and rage, and he described a scene that, whether experienced or not, was truer than any description of what actually happened. Pale and upset, he explained that his alter ego would have been “beaten up by this strong and solid uncle. That he was strangled in his parents’ house, in front of them. That he fell on the floor when his uncle finally released him”, and that this would be a final break with his adoptive family. It was a humiliation that he clearly still resented today, but that we were able to integrate into our narrative because it was co-written and fictionalized, thereby infusing the life history with much more emotional depth than it would have had otherwise.

## Conclusion

Co-writing with a gang member extended my ethnographic fieldwork. It improved my understanding of my own biases, as well as of my research interlocutor Dino’s moral and ethical grounding. What is interesting is that I discovered how this ethic and rules constrained my interlocutors’ ability to tell his own story. His moral views and ethics would prevail over the academic standards that I was trying to uphold, what is often called “research integrity” but actually often has more to do with presentational issues. Our solution was to fictionalize our co-written life history and make it about a carefully crafted alter ego of Dino’s, something that generated a number of new research insights. The process gave me access to Dino’s rewritings and “corrections” of what he told me in our interviews, as well as a whole range of stories about gang lives. These blurred the differences between lived and imagined experience, reality and fiction. While all ethnographers face issues of manipulation or misrepresentation, and ethnographic writing involves an unacknowledged element of fictionalization, it was the gaps, half-truths, and inventions in Dino’s narrative about his life that generated the most insightful material for analysis.

Co-writing also established a fairer ethnographic “pact”. It forced me to confront questions I tended to avoid about my own biases and about what we deem interesting to research. Because we want to advance an argument, because we believe we should focus on a specific dimension of gangs, or because we are influenced by canonical literature and other representations on gangs, we may miss or ignore the “essence” of the stories our interlocutors want to tell. At the same time, co-writing a life story with a former gangster raises a range of ethical issues. This was well formulated by Leigh Payne in an ERC GANGS project workshop in Oxford, when she asked me, in response to my presenting an early iteration of this paper, “but what is the role of the ethnographer here, and can co-writing be done with all issues and people?”. With regard to the former issue, part of the reason I was able to engage in this co-writing process with Dino was that I had enough knowledge and experience to interpret, contextualize, and sometimes challenge Dino’s narratives, pushing him to think outside his established frameworks and understandings, and to reflect on the evolution of his own interpretations and ideas. This simply would not have been possible without this background, which is something that distinguishes ethnographers from most journalists or investigators, for example. At the same time, it would equally have been impossible to truly get to grips with the gangster experience if I haven’t had access to Dino, to understand how he thought and interpreted the world around him, tried to give meaning to his situation, and how being a gang member impacted his life. Ultimately, then, our roles were symbiotic.

With regard to the latter issue, whether co-writing can be done with all issues and people, I believe the answer is no. Putting aside obvious issues such as literacy, etc., sometimes it is clear that power differences can become significant obstacles. Indeed, this seemed to initially be the case between Dino and myself, until we established our new ethnographic pact that allowed us to co-write his life story. But there can also be epistemological issues that are more difficult to surmount, as the single authored nature of this article attests. Despite the establishment of a new ethnographic pact, there are some forms of knowledge production and writing that remain distant from the societies and people we research. On the one hand, I undertook to write it alone because I did not have the opportunity to discuss it with Dino in the same way as we discussed our co-written fictionalized life history. On the other hand, this article is an unexpected output of the latter experiment, one that came about after I had finished interviewing and co-writing with Dino, in response to questions that I received from ERC GANGS project participants asking me what I had got out of the co-writing process, epistemologically-speaking. It’s an article that has been written for a different audience than the fictionalized life history co-written with Dino, offering the reflections of a social scientist about how the latter process changed him and his interpretations. Dino may disagree with some of my views and understandings that I have written here, but they are also something that he may be less interested in, because it is not his story. And in relation to the latter, not only did our collaboration allow us to deal with power and epistemological differences, but it was ultimately also the only way to write a fair story.

## Footnotes

1 This name, as per the wishes of Dino, is a pseudonym.

2 See https://www.imdb.com/title/tt2375605/.
